# Association of Frailty and Malnutrition With Long-term Functional and Mortality Outcomes Among Community-Dwelling Older Adults: Results From the Singapore Longitudinal Aging Study 1

**DOI:** 10.1001/jamanetworkopen.2018.0650

**Published:** 2018-07-13

**Authors:** Kai Wei, Ma-Shwe-Zin Nyunt, Qi Gao, Shiou-Liang Wee, Keng-Bee Yap, Tze-Pin Ng

**Affiliations:** 1 Geriatric Education and Research Institute, Singapore; 2 currently with Shanghai Center for Systems Biomedicine, Shanghai Jiao Tong University, Shanghai, China; 3 Gerontology Research Programme, Department of Psychological Medicine, National University of Singapore, Singapore; 4 Faculty of Health and Social Sciences, Singapore Institute of Technology, Singapore; 5 Department of Geriatric Medicine, Ng Teng Fong General Hospital, Singapore

## Abstract

**Importance:**

Physical frailty and malnutrition are prevalent among older adults and may be associated with functional and mortality outcomes.

**Objective:**

To assess the health outcomes associated with physical frailty and malnutrition singly and in combination among older adults.

**Design, Setting, and Participants:**

Population-based cohort study (Singapore Longitudinal Aging Study 1). Included were 2804 community-dwelling adults in Singapore aged 55 years or older at baseline (September 1, 2003, to December 23, 2005), with 2 follow-ups at 2- to 3-year intervals (from March 7, 2005, to September 10, 2007, and from November 13, 2007, to December 12, 2009) and a 12-year mortality follow-up to March 31, 2017. Data analysis was from July 1 to September 28, 2017.

**Main Outcomes and Measures:**

Baseline physical frailty (Fried criteria) with participants categorized according to the total score as frail (3-5 points), prefrail (1-2 points), or robust (0 point), and nutritional status (Nutrition Screening Initiative DETERMINE Your Nutritional Health Checklist and Mini Nutritional Assessment Short-Form [MNA-SF]). Baseline (prevalent) and follow-up (incident) instrumental/basic activities of daily living (IADL/ADL) disability, poor quality of life (QOL), and mortality were measured. Estimates of association were by odds ratios (ORs) and hazard ratios (HRs) and their 95% CIs.

**Results:**

The participants (mean [SD] age, 66.0 [7.7] years; 1033 [36.8%] male; 2611 [93.1%] Chinese) included 1021 (37.6%) categorized as robust with MNA-SF normal nutrition (R-NN), 330 (12.2%) robust with MNA-SF at risk/malnourished (R-ARM), 734 (27.0%) prefrail/frail with MNA-SF normal nutrition (PFF-NN), and 631 (23.2%) prefrail/frail with MNA-SF at risk/malnourished (PFF-ARM). Among these 2804 participants, 44 had missing frailty status, and 78 had missing MNA-SF nutritional status; therefore, 88 participants in total had missing frailty-nutritional status. In cross-sectional analyses, the prevalence of IADL/ADL disability was lowest among the R-NN group (169 [16.9%]) and increased substantially only among the PFF-ARM group (249 [40.2%]) (OR, 1.88; 95% CI, 1.40-2.53). Poor QOL prevalence was lowest among the R-NN group (142 [14.1%]), and the increase in other frailty and nutritional status groups was highest in the PFF-ARM group (255 [41.3%]) (OR, 2.61; 95% CI, 1.96-3.49). In longitudinal analyses, significant association with only incident poor QOL across frailty and nutritional status groups was highest in the PFF-ARM group (89 [34.8%]) compared with the R-NN group (132 [19.2%]) (OR, 1.70; 95% CI, 1.17-2.48). The mortality rate was lowest in the R-NN group (0.54 per 100 person-years) and highest in the PFF-ARM group (3.04 per 100 person-years) (HR, 1.72; 95% CI, 1.01-2.92). The results based on the Nutrition Screening Initiative measure of nutritional status were similar.

**Conclusions and Relevance:**

Reported adverse health outcomes attributed to poor nutrition often appear more likely to be associated with physical frailty. Prefrail/frail older persons with poor nutrition might be targeted for interventions to prevent or delay adverse functional and mortality outcomes.

## Key Points

**Question:**

How are physical frailty and malnutrition singly and in combination associated with adverse health outcomes?

**Findings:**

In this cohort study of 2804 community-dwelling adults in Singapore, poor nutrition alone without physical frailty was not significantly associated with increased prevalence or incidence of functional disability, poor quality of life, or mortality, while poor nutrition with prefrailty/frailty was consistently associated with substantially increased prevalence and incidence of poor functional and mortality outcomes.

**Meaning:**

Reported adverse health outcomes attributed to poor nutrition often appear more likely to be associated with physical frailty; therefore, prefrail/frail older persons with poor nutrition might be targeted for interventions to prevent or delay adverse health outcomes.

## Teaser

This population-based cohort study assesses the adverse health outcomes associated with physical frailty and malnutrition singly and in combination among older adults.

## Introduction

A common condition of old age is malnutrition, which results from inadequate food intake, unmet increased protein and calorie demand, chronic diseases, polypharmacy, or functional disability. Malnutrition is prevalent among approximately one-third of older people, especially residents in institutionalized facilities.^1^ Malnutrition is associated with increased risks of many adverse health outcomes, including longer length of hospitalization stay, higher prevalence of comorbidities (eg, infections and fractures), increased severity of disability, decreased quality of life (QOL), and higher mortality rates.^2,3,4,5,6^

Frailty is a common geriatric syndrome reflecting an underlying condition of depleted functional reserve owing to underlying multisystem physiological dysregulation, rendering the older persons vulnerable to the effects of stress and at risk of adverse health outcomes, such as functional disability, hospitalization, poor QOL, and mortality. Physical frailty may result not only from malnutrition^7^ but also from other causes, such as physical inactivity, chronic diseases, polypharmacy, and hormonal, cytokine, and metabolic imbalances.^8^

Although they are distinct conditions, physical frailty and malnutrition are closely associated.^9,10,11,12^ The 2 conditions share many common pathophysiological pathways, including the loss of body tissue and chronic inflammation, and common sociodemographic, physical, and cognitive risk factors, including functional disability.^10,12^ Hence, it is not uncommon to encounter older individuals with both physical frailty and malnutrition.^7,8^ According to evidence, 2 of 3 malnourished older adults were physically frail (based on a Fried physical phenotype criteria score of 3-5); approximately 1 in 10 of the physically frail population was malnourished (by Mini Nutritional Assessment Short-Form [MNA-SF] criteria).^11^ In a previous population study of older Singaporeans,^10^ the overall prevalence of MNA-SF malnutrition (2.8%) and at risk of malnutrition (27.6%) was 30.4% in total; the prevalence of frailty (4.5%) and prefrailty (46.0%) was 50.5% in total. Among malnourished individuals, the prevalence of frailty (26.5%) and prefrailty (64.2%) was 90.7% in total. Among frail older persons, the prevalence of malnutrition was 16.1%; the proportion increased to two-thirds if those who were at risk of malnutrition were included.

Previous studies^3,6^ have investigated the association of malnutrition with adverse outcomes without considering the presence of frailty. To our knowledge, no studies have reported that co-occurring frailty and malnutrition are associated with higher rates of functional disability, poor QOL, or mortality than either alone. In this population-based cohort study, we assessed the adverse health outcomes associated with physical frailty and malnutrition (singly and in combination) among 2804 community-dwelling older adults in the Singapore Longitudinal Aging Study 1 (SLAS-1) cohort. We examined the prevalence of co-occurring physical frailty and malnutrition/nutritional risk, and we investigated the associations of co-occurring physical frailty and malnutrition/nutritional risk with prevalent and incident functional disability, poor QOL, and mortality in cross-sectional and longitudinal analyses.

## Methods

### Participants

We analyzed data collected from the first-wave cohort (SLAS-1) of the Singapore Longitudinal Aging Study. As previously described,^13,14,15^ the SLAS is a population-based longitudinal study of aging and health of community-dwelling Singaporeans 55 years or older at baseline, excluding individuals who were not able to participate because of severe physical or mental disability. The first cohort (SLAS-1) recruited 2804 residents in the southeast region of Singapore between September 1, 2003, and December 23, 2005, with 2 follow-ups at approximately 2- to 3-year intervals from March 7, 2005, to September 10, 2007, and from November 13, 2007, to December 12, 2009, as well as a 12-year mortality follow-up to March 31, 2017. Data analysis was conducted from July 1 to September 28, 2017. The study received ethical approval from the National University of Singapore Institutional Review Board. Written informed consent was obtained from all participants. This study followed the Strengthening the Reporting of Observational Studies in Epidemiology (STROBE) reporting guideline.

### Measurements

In this study, we used baseline data of physical frailty and nutritional status and assessed their associations with prevalent and incident adverse health outcomes. The latter included cumulative incident cases of functional dependency and poor QOL assessed from the 2 follow-up visits and deaths determined from baseline to March 31, 2017.

#### Frailty

The physical frailty phenotype was assessed at baseline based on the 5 criteria proposed by Fried et al^16^ in the Cardiovascular Health Study. Because the knee extension measure of muscle strength and the fast gait speed test of gait speed were only available in a subsequent cohort (SLAS-2), we used 5 operationally modified measures of weakness and slowness in this study. First, shrinking was defined as body mass index (calculated as weight in kilograms divided by height in meters squared) of less than 18.5 and/or unintentional weight loss of at least 4.5 kg (10 lb) in the past 6 months. Second, weakness was defined as the lowest quintile of performance on the rising from chair test in the sitting position with arms folded based on the Performance Oriented Mobility Assessment (POMA) battery.^17^ Third, slowness was assessed by POMA gait tests (participants walked 6 m and returned to the starting point quickly),^17^ which include 7 gait items (initiation of gait, step length and height, step symmetry, step continuity, and path, trunk, and walking stance). The total POMA gait score has a range from 0 to 12, and a score of less than 9 denotes slowness. Fourth, exhaustion was determined by response of “not at all” to the following question from the Medical Outcomes Study 12-Item Short Form Health Survey (SF-12) QOL scale: “Do you have a lot of energy?” Fifth, low activity was determined by self-report of “none” for participation in any physical activity (walking or recreational or sports activity).

One point was assigned for the presence of each of the 5 components, and participants were categorized according to the total score as frail (3-5 points), prefrail (1-2 points), or robust (0 point). Previous studies^13,14,15,18^ have shown that this modified version of the physical frailty index has fair to good agreement (weighted κ = 0.63) with versions based on the knee extension measure of muscle strength and the 6-m fast gait speed test and was equally and strongly determinant of adverse health outcomes, including cognitive impairment, instrumental/basic activities of daily living (IADL/ADL) dependency, hospitalization, and poor QOL.

#### Nutritional Status

At baseline, nutritional status was assessed using the Nutrition Screening Initiative (NSI) and the MNA-SF. The NSI, also called DETERMINE Your Nutritional Health Checklist,^19^ is used to assess nutritional risk based on a 10-item questionnaire describing personal and behavioral factors associated with inadequate or poor-quality food and nutrient intake among older persons. The total weighted NSI score ranges from 0 to 21; a score of 6 or higher indicates high nutritional risk, 3 to 5 indicates moderate nutritional risk, and 0 to 2 indicates good nutritional status.

The MNA-SF^20,21^ is a widely used nutrition screening scale. We derived the MNA-SF score from available data collected in the SLAS-1 cohort as previously reported.^10^ The total weighted MNA-SF score ranges from 0 to 14; a score of 12 to 14 indicates normal nutritional status, 8 to 11 indicates at risk of malnutrition, and 7 or lower indicates malnourished.

#### Adverse Health Outcomes

The main adverse health outcomes assessed in our study included IADL/ADL disability, poor QOL assessed from the 2 follow-up visits, and mortality. The IADL/ADL disability was determined by self-reported difficulty and/or requiring assistance in at least 1 IADL and/or ADL activity from the Lawton Instrumental Activity of Daily Living and Barthel Basic Activities of Daily Living instruments. Quality of life was measured using the SF-12 physical component score and mental component score scales, and poor QOL was indicated by values below the lowest quartile of physical component score. Mortality data until March 31, 2017, were obtained from computerized national record linkage with the National Death Registry through the Singapore National Registry of Diseases Office.

#### Covariates

Sociodemographic data included age, sex, education, housing type (an indicator of socioeconomic status), race/ethnicity, marital status, and living arrangement. Self-report of a medical disorder diagnosed and treated by a physician was recorded for 22 named diagnoses and other disorders. The number of comorbidities was estimated from the total count of medical disorders in the past year. Polypharmacy was defined as the use of 5 or more medications. Cognitive function was assessed using the Mini-Mental State Examination, with a maximum score of 30; a score of 24 or higher indicates normal cognition, 19 to 23 indicates mild cognitive impairment, and 18 or lower indicates moderate to severe cognitive impairment. Depressive symptoms were measured by the Geriatric Depression Scale (GDS), which has been validated for use in Singaporeans.^22,23^ With a maximum score of 15, a GDS score of 5 or higher indicates clinically significant depression. Hospitalization was determined by self-report of new hospitalizations for any medical conditions in the past year.

### Statistical Analysis

Categorical variables are presented as numbers (percentages), while continuous variables are presented as means (SDs). Differences in the distribution of categorical variables among groups were tested by **χ**^2^ test. For continuous variables, the *F* test or Kruskal-Wallis test was used for comparison between different groups. Logistic regression was performed to calculate odds ratios (ORs) (95% CIs) of association between baseline frailty and nutritional status and prevalent functional disability and poor QOL in cross-sectional analyses, as well as cumulative incident cases of functional disability and poor QOL determined from both follow-up visits in longitudinal analyses. Cox proportional hazards regression that satisfied the proportional hazard assumption was performed to calculate hazard ratios (HRs) (95% CIs) of association between frailty and nutritional status and mortality, and Kaplan-Meier survival curves were plotted to compare the survival rates up until March 31, 2017, among different groups. Estimated ORs and HRs were adjusted for the following model covariates: age, sex, education, housing type, race/ethnicity, marital status, current smoking, alcohol drinking, comorbidities, polypharmacy, type 2 diabetes, anemia, chronic obstructive pulmonary disease, hip fracture, cardiac diseases, chronic kidney disease, history of stroke, visual impairment, cognitive impairment, depressive syndromes, and hospitalization. Because the number of participants in the malnourished or high nutritional risk groups were small when categorized with frailty status, these participants were combined with the at risk of malnutrition or moderate nutritional risk groups in the analyses. An acceptable level of significance was established as 2-sided *P* < .05. Stata 12.0 (StataCorp LP) was used for data analysis.

## Results

The mean (SD) age of the 2804 study participants was 66.0 (7.7) years (age range, 55-98 years), 1033 (36.8%) were male, 1465 (52.2%) had primary or lower education, 1755 (62.6%) lived in public housing of 3 to 5 rooms, and 2611 (93.1%) were Chinese. Among all the participants, 1370 (49.6%) were robust, 1292 (46.8%) were prefrail, and 98 (3.6%) were frail. The MNA-SF showed that 1762 (64.6%) had normal nutritional status, 857 (31.4%) were at risk of malnutrition, and 107 (3.9%) were malnourished. The NSI DETERMINE Checklist indicated that 1945 (69.6%) had good nutrition, 718 (25.7%) had moderate nutritional risk, and 131 (4.7%) had high nutritional risk (Table 1). By frailty and nutritional status, 1021 (37.6%) were robust with MNA-SF normal nutrition, 330 (12.2%) were robust with MNA-SF at risk/malnourished, 734 (27.0%) were prefrail/frail with MNA-SF normal nutrition, 631 (23.2%) were prefrail/frail with MNA-SF at risk/malnourished, and 521 (18.9%) were prefrail/frail with NSI moderate/high nutritional risk (Table 2). The numbers of participants with complete and missing data at baseline and follow-ups are listed in eTable 1 in the Supplement. Among these 2804 participants, 44 had missing frailty status, and 78 had missing MNA-SF nutritional status; therefore, 88 participants in total had missing frailty-nutritional status.

**Table 1.** Sociodemographic Characteristics and the Prevalence of Frailty and Malnutrition/Nutritional Risk Among the Singapore Longitudinal Aging Study 1 Cohort

Variable	Value
No. of participants	2804
Age, mean (SD), y	66.0 (7.7)
Male, No. (%)	1033 (36.8)
Education, No. (%)	
No education	538 (19.2)
Primary	927 (33.1)
Secondary/higher	1339 (47.8)
Housing type, No. (%)	
Public housing of 1-2 rooms	171 (6.1)
Public housing of 3-5 rooms	1755 (62.6)
High-end public and private housing	878 (31.3)
Race/ethnicity, No. (%)	
Chinese	2611 (93.1)
Malay	104 (3.7)
Indian/others	89 (3.2)
Single/divorced/widowed, No. (%)	736 (26.2)
Living alone, No. (%)	201 (7.2)
Physical frailty, No. (%)	(n = 2760)
Robust	1370 (49.6)
Prefrail	1292 (46.8)
Frail	98 (3.6)
MNA-SF, No. (%)	(n = 2726)
Normal nutrition	1762 (64.6)
At risk of malnutrition	857 (31.4)
Malnourished	107 (3.9)
NSI, No. (%)	(n = 2794)
Good nutrition	1945 (69.6)
Moderate nutritional risk	718 (25.7)
High nutritional risk	131 (4.7)

Abbreviations: MNA-SF, Mini Nutritional Assessment Short-Form; NSI, Nutrition Screening Initiative.

**Table 2.** Total Population Sample Numbers and Proportions of Participants With Malnutrition/Nutritional Risk

Nutritional Status Score Range	No. (%)				Nutritional Status	No. (%)	
	Total	Robust	Prefrail	Frail		Robust	Prefrail/Frail
MNA-SF score	2716 (100)	1351 (49.7)	1271 (46.8)	94 (3.5)	MNA-SF	1351 (49.7)	1365 (50.3)
Normal nutrition, 12-14	1755 (64.6)	1021 (75.6)	715 (56.3)	19 (20.2)	Normal nutrition	1021 (37.6)	734 (27.0)
At risk of malnutrition, 8-11	855 (31.5)	320 (23.7)	487 (38.3)	48 (51.1)	At risk/malnourished	330 (12.2)	631 (23.2)
Malnourished, 0-7	106 (3.9)	10 (0.7)	69 (5.4)	27 (28.7)			
NSI score	2754 (100)	1367 (49.6)	1290 (46.8)	97 (3.5)	NSI	1367 (49.6)	1387 (50.4)
Good nutrition, 0-2	1918 (69.6)	1052 (77.0)	826 (64.0)	40 (41.2)	Good nutrition	1052 (38.2)	866 (31.4)
Moderate nutritional risk, 3-5	707 (25.7)	278 (20.3)	388 (30.1)	41 (42.3)	Moderate/high nutritional risk	315 (11.4)	521 (18.9)
High nutritional risk, ≥6	129 (4.7)	37 (2.7)	76 (5.9)	16 (16.5)			

Abbreviations: MNA-SF, Mini Nutritional Assessment Short-Form; NSI, Nutrition Screening Initiative.

### Physical Frailty/Nutritional Status and Adverse Health Outcomes

**Figure 1.**
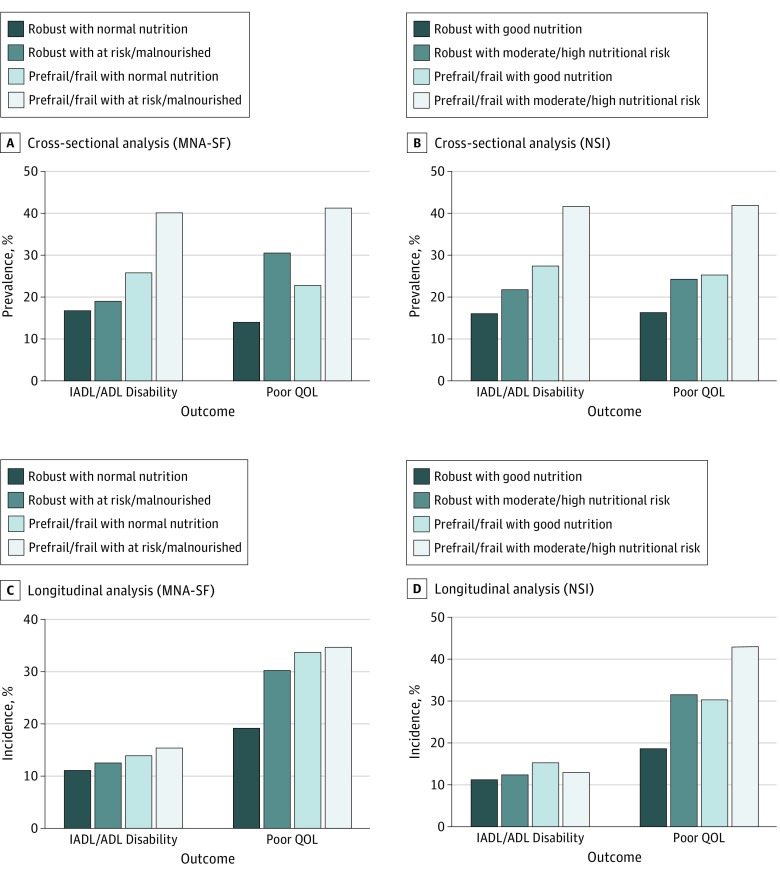
Prevalence and Incidence of Adverse Health Outcomes According to Baseline Frailty-Nutritional Status A, Prevalence of instrumental/basic activities of daily living (IADL/ADL) disability and poor quality of life (QOL) according to baseline frailty and nutritional status (Mini Nutritional Assessment Short-Form [MNA-SF]). B, Prevalence of IADL/ADL disability and poor QOL according to baseline frailty and nutritional status (Nutrition Screening Initiative [NSI]). C, Incidence of IADL/ADL disability and poor QOL according to baseline frailty and nutritional status (MNA-SF). D, Incidence of IADL/ADL disability and poor QOL according to baseline frailty and nutritional status (NSI).

Table 3 summarizes the results of analyses of the associations of baseline groups of frailty and nutritional status (by MNA-SF and NSI) with prevalent and incident adverse health outcomes, including IADL/ADL disability, poor QOL, and mortality. The estimates of association were adjusted for variables known to be associated with physical frailty and malnutrition, including age, sex, education, housing type, race/ethnicity, marital status, current smoking, alcohol drinking, comorbidities, polypharmacy, type 2 diabetes, anemia, chronic obstructive pulmonary disease, hip fracture, cardiac diseases, chronic kidney disease, history of stroke, visual impairment, cognitive impairment, depressive syndromes, and hospitalization. Robust with MNA-SF normal nutrition (or robust with NSI good nutrition) was used as the reference group in the logistic regression and Cox proportional hazards regression analyses. Overall, compared with frailty status alone or malnutrition and nutritional risk status alone, the co-occurrence of frailty and poor nutritional status was associated with additional greater risks for adverse health outcomes at baseline and follow-ups (Figure 1). The incidences of adverse health outcomes according to baseline frailty and nutritional status are listed in eTable 2 in the Supplement.

**Table 3.** Baseline Malnutrition/Nutritional Risk Status With Prevalent and Incident Adverse Health Outcomes by Frailty Phenotype^a^

Variable	IADL/ADL Disability			Poor QOL			Mortality^b^		
	No. (%)	OR (95% CI)^c^	*P* Value	No. (%)	OR (95% CI)^c^	*P* Value	Per 100 Person-Years	HR (95% CI)^c^	*P* Value
**Cross-sectional Analyses**									
Frailty status, MNA-SF, total No.	2669			2682					
Robust									
Normal nutrition	169 (16.9)	1 [Reference]	NA	142 (14.1)	1 [Reference]	NA	NA	NA	NA
At risk/malnourished	61 (19.0)	1.04 (0.72-1.51)	.84	100 (30.5)	2.02 (1.45-2.81)	<.001	NA	NA	NA
Prefrail/frail									
Normal nutrition	187 (25.8)	1.22 (0.94-1.59)	.14	166 (22.8)	1.45 (1.12-1.90)	.006	NA	NA	NA
At risk/malnourished	249 (40.2)	1.88 (1.40-2.53)	<.001	255 (41.3)	2.61 (1.96-3.49)	<.001	NA	NA	NA
Frailty status, NSI, total No.	2701			2701					
Robust									
Good nutrition	166 (16.1)	1 [Reference]	NA	168 (16.3)	1 [Reference]	NA	NA	NA	NA
Moderate/high nutritional risk	67 (22.0)	1.08 (0.76-1.54)	.66	76 (24.3)	1.26 (0.90-1.77)	.18	NA	NA	NA
Prefrail/frail									
Good nutrition	234 (27.5)	1.29 (1.00-1.67)	.05	215 (25.4)	1.38 (1.07-1.76)	.01	NA	NA	NA
Moderate/high nutritional risk	213 (41.6)	1.88 (1.39-2.52)	<.001	213 (41.8)	2.06 (1.55-2.75)	<.001	NA	NA	NA
**Longitudinal Analyses^d^**									
Frailty status, MNA-SF, total No.	1521			1525			2500		
Robust									
Normal nutrition	73 (11.1)	1 [Reference]	NA	132 (19.2)	1 [Reference]	NA	0.54	1 [Reference]	NA
At risk/malnourished	23 (12.6)	1.01 (0.57-1.78)	.98	51 (30.2)	1.44 (0.92-2.23)	.11	0.86	0.81 (0.39-1.70)	.58
Prefrail/frail									
Normal nutrition	56 (13.9)	1.08 (0.72-1.62)	.71	139 (33.7)	1.58 (1.16-2.14)	.003	1.34	1.35 (0.79-2.29)	.27
At risk/malnourished	42 (15.3)	0.98 (0.61-1.59)	.94	89 (34.8)	1.70 (1.17-2.48)	.006	3.04	1.72 (1.01-2.92)	.04
Frailty status, NSI, total No.	1533			1534			2533		
Robust									
Good nutrition	74 (11.2)	1 [Reference]	NA	125 (18.7)	1 [Reference]	NA	0.50	1 [Reference]	NA
Moderate/high nutritional risk	23 (12.3)	0.82 (0.47-1.43)	.48	60 (31.6)	1.31 (0.87-1.96)	.19	0.96	1.09 (0.55-2.19)	.80
Prefrail/frail									
Good nutrition	72 (15.4)	1.09 (0.75-1.61)	.64	141 (30.2)	1.45 (1.07-1.96)	.02	1.78	1.63 (0.98-2.71)	.06
Moderate/high nutritional risk	28 (13.0)	0.76 (0.45-1.30)	.32	89 (43.0)	1.97 (1.34-2.89)	.001	2.77	1.86 (1.08-3.19)	.02

Abbreviations: HR, hazard ratio; IADL/ADL, instrumental/basic activities of daily living; MNA-SF, Mini Nutritional Assessment Short-Form; NA, not applicable; NSI, Nutrition Screening Initiative; OR, odds ratio; QOL, quality of life.

^a^ The denominators for the percentages were the total number of participants in each frailty-nutritional group (eg, the prevalence of 16.9% meant that 169 among the 1003 participants in the robust with MNA-SF normal nutrition group had IADL/ADL disability at baseline).

^b^ Cox proportional hazards regression was used to calculate the HR of mortality.

^c^ Adjusted for age, sex, education, housing type, race/ethnicity, marital status, current smoking, alcohol drinking, comorbidities, polypharmacy, type 2 diabetes, anemia, chronic obstructive pulmonary disease, hip fracture, cardiac diseases, chronic kidney disease, history of stroke, visual impairment, cognitive impairment, depressive syndromes, and hospitalization.

^d^ Longitudinal analyses were performed in individuals without baseline IADL/ADL disability or poor QOL for the respective outcomes.

### IADL/ADL Disability

In cross-sectional analyses, the group of robust with MNA-SF normal nutrition had the lowest prevalence of IADL/ADL disability (169 [16.9%]), other groups that included the presence of prefrailty/frailty or MNA-SF at risk/malnourished had a higher prevalence of IADL/ADL disability, and the group of prefrail/frail with MNA-SF at risk/malnourished had the highest prevalence of IADL/ADL disability (249 [40.2%]) (OR, 1.88; 95% CI, 1.40-2.53). Similar results were observed for groups of robust with NSI good nutrition and prefrail/frail with NSI moderate/high nutritional risk (OR, 1.88; 95% CI, 1.39-2.52). However, in longitudinal analyses, baseline co-occurring prefrail/frail with nutritional risk (MNA-SF or NSI) were not found to be significantly associated with increased risks of incident IADL/ADL disability.

### Poor QOL

In contrast, co-occurring prefrail/frail with nutritional risk (MNA-SF or NSI) was significantly associated with poor QOL in both cross-sectional and longitudinal analyses. Poor QOL prevalence was lowest among the robust with MNA-SF normal nutrition group (142 [14.1%]), and the increase in other frailty and nutritional status groups was highest in the prefrail/frail with MNA-SF at risk/malnourished group (255 [41.3%]) (OR, 2.61; 95% CI, 1.96-3.49). In longitudinal analyses, significant association with only incident poor QOL across frailty and nutritional status groups was highest in the prefrail/frail with MNA-SF at risk/malnourished group (89 [34.8%]) compared with the robust with MNA-SF normal nutrition group (132 [19.2%]) (OR, 1.70; 95% CI, 1.17-2.48). Similar magnitudes of increases in prevalence and incidence of poor QOL were observed from under 20% in the robust with MNA-SF normal nutrition (or robust with NSI good nutrition) group to 35% to 43% in the prefrail/frail with MNA-SF at risk/malnourished (or prefrail/frail with NSI moderate/high nutritional risk) groups, with statistically significant 2-fold (95% CI range, 1.17-3.49; *P* < .01 for all) increases in poor QOL compared with robust with MNA-SF normal nutrition or robust with NSI good nutrition. The group of prefrail/frail with MNA-SF normal nutrition (or NSI good nutrition) was also associated with statistically significant, approximately 1.5-fold (95% CI range, 1.07-2.14; *P* < .05 for all) elevated prevalence and incidence of poor QOL.

### Mortality

**Figure 2.**
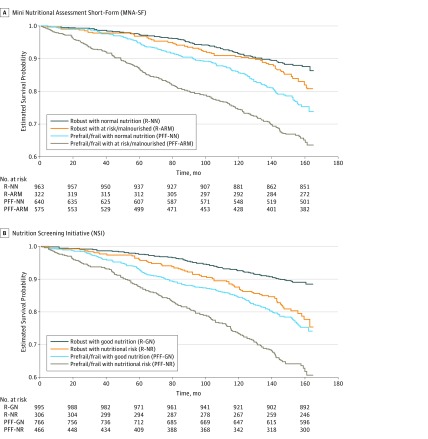
Kaplan-Meier Survival Curves of Older Adults in the Singapore Longitudinal Aging Study 1 Cohort Kaplan-Meier survival curves were plotted to compare the survival rates up until March 31, 2017, according to baseline frailty and nutritional status. A, Kaplan-Meier survival curve according to baseline frailty and nutritional status (MNA-SF, n = 2500; *P* < .001). B, Kaplan-Meier survival curve according to baseline frailty and nutritional status (NSI, n = 2533; *P* < .001).

The mortality risk increased from 0.54 per 100 person-years to 3.04 per 100 person-years across the 4 frailty and MNA-SF nutrition groups, including robust with MNA-SF normal nutrition, robust with MNA-SF at risk/malnourished, prefrail/frail with MNA-SF normal nutrition, and prefrail/frail with MNA-SF at risk/malnourished. The estimated mortality HR associated with co-occurring prefrail/frail and poor nutrition (both MNA-SF and NSI) indicated increased mortality risk (HR, 1.72; 95% CI, 1.01-2.92 for MNA-SF and HR, 1.86; 95% CI, 1.08-3.19 for NSI; *P* < .05 for both) compared with robust with MNA-SF normal nutrition or robust with NSI good nutrition. The survival curves across the 4 groups are shown in Figure 2.

## Discussion

There is little understanding of the natural histories of physical frailty and malnutrition with respect to each other, particularly in their order of appearance and codevelopment in old age. One can occur before the other, as evidenced by the presence of one without the other in this study. The presence of malnutrition can subsequently lead to frailty, and the presence of frailty can lead to malnutrition, culminating in a pool of older persons who are both frail and malnourished. Malnutrition or being at risk of malnutrition may subsequently result in prefrailty/frailty, and limited evidence supports this possibility.^24,25^ Conversely, it is plausible that prefrailty/frailty can be present without initial evidence of malnutrition, possibly owing to many other causes, and prefrailty/frailty can be the cause of concurrent or subsequent malnutrition through both intrinsic pathophysiological and extrinsic psychosocial mechanisms. To our knowledge, no follow-up studies have shown this temporal occurrence, although the results of cross-sectional studies (eg, the 2017 study by Wei et al^10^), including the present study, suggest that this temporality is a likely possibility. Limited evidence from our study indicates that, regardless of whether physical frailty or malnutrition occurs first, there is possibly a closed-loop cyclical association between the two in progressing toward a combined frailty-malnutrition state. This association is reflected by the progressive increase in the prevalence of frailty across MNA-SF normal nutrition and at risk and malnourished groups (and NSI good nutrition and moderate and high nutritional risk groups), as well as the progressive increase in MNA-SF malnutrition (or NSI high nutritional risk) prevalence from robust to prefrail to frail groups. However, this finding should be further investigated in prospective follow-up studies.

While previous studies^10,12^ have shown that physical frailty and malnutrition are both associated with increased risk of adverse health outcomes, the present study is the first, to our knowledge, that has assessed their individual and combined associations with adverse health outcomes. The results of our study suggest that, in the absence of physical frailty, poor nutrition was associated with only a nonsignificant small increase in adverse functional and mortality outcomes, whereas physical frailty in the absence of poor nutrition was associated with a moderately greater increase in risks of poor functional outcomes, especially poor QOL and mortality. In contrast, the co-occurrence of physical frailty and poor nutrition was associated with a much greater increase in adverse health outcomes. These findings indicate that the results of adverse consequences of malnutrition reported in previous studies^2,3,4,5,6^ could occur because of the unreported presence of physical frailty. Our study findings also suggest that physical frailty itself appears to be a stronger determinant than malnutrition of adverse functional and mortality outcomes, as well as a likely mediating cause of the adverse health outcomes associated with malnutrition. However, we did not collect follow-up data on frailty status in this cohort to investigate this association.

### Practical Implications of the Study Findings

Health care systems in aging and aged societies face major challenges in solving the problem of growing and large numbers of frail older persons who are dependent on others in performing ADL. There is increasing recognition that the prevention and treatment of physical frailty is a key strategy to preventing or delaying the onset of functional disability and improving QOL among older people. Evidence supports recommendations for the screening, assessment, and treatment of physical frailty and malnutrition in clinical, institutional, and community settings to prevent future disability, hospitalizations, and institutionalization.^26,27^ Simple and validated screening tools for frailty (eg, the FRAIL scale^28^) and malnutrition (eg, the NSI) are available, and evidence has shown frailty to be reversible by nutritional, physical, and cognitive interventions and medical management (eg, decreasing polypharmacy).^29^ Our findings have particular relevance for population-based strategies in identifying prefrail and nutritionally at risk older people who could benefit from general public health education and community-based lifestyle interventions to regain physical robustness and good nutrition and arrest progression to frailty and malnutrition. A particularly high-risk group identified in this study is older persons who are concurrently prefrail/frail and nutritionally at risk/malnourished, who should be targeted for clinical interventions. These people represent approximately 20% of the population in our Asian study, consistent with other similar estimates.^11^

### Limitations of Nutritional Status Assessment

In this study, we chose to use the MNA-SF and the NSI in parallel to assess nutritional status. There is no ideally accurate measurement tool for malnutrition.^30^ Although the MNA-SF is the most widely used instrument for screening and assessing nutritional status, it should be noted that two-thirds of its items, including weight loss and immobility, are closely associated with physical frailty.^9,31^ In using the MNA-SF, there is thus some degree of conflation between frailty and malnutrition assessments. In particular, the only positive association between poor nutrition in the absence of physical frailty with a higher prevalence of poor QOL was observed with the use of MNA-SF at risk/malnourished status to assess poor nutrition and not with NSI moderate/high nutritional risk status. Compared with the MNA-SF, the NSI has less phenotypic overlap with physical frailty. The NSI nutritional risk index carries greater weight of food intake–related risk factors, especially inadequate dietary intake and nutritional deficiency owing to change of eating behavior and socioeconomic status.^5^ The effect size associated with the MNA-SF measure of nutrition appears to be overestimated relative to the NSI measure; nevertheless, similar associations were observed with both in our study.

### Strengths and Limitations

A particular strength of this study is that we were able to ascertain the long-term associations between physical frailty and nutritional status and future adverse functional outcomes over 5 to 6 years and mortality up to 12 years of follow-up. As mentioned earlier, a limitation of the present study is that physical frailty was only assessed at baseline (between September 1, 2003, and December 23, 2005); therefore, we could not investigate the dynamic temporal associations of physical frailty and nutritional status through the course of the study. It is likely that there were unmeasured changes in the frailty status and nutritional status of participants over time that could also obscure the observed associations in an unknown fashion. In addition, because participants who were frailer at baseline were more likely to be lost to follow-up, the study was biased by selecting participants who were healthier at baseline, and this factor may possibly have weakened the effect size for adverse health outcomes.

## Conclusions

Reported adverse health outcomes attributed to poor nutrition often appear more likely to be associated with physical frailty. Further research should be conducted to better understand the dynamic temporal associations between frailty and malnutrition in their order of appearance and codevelopment. Interventional studies should investigate the associations of intervening on frailty and nutritional status to produce favorable health, functional, and mortality outcomes.

## Notes

Supplement. **eTable 1.** The Number of Participants With Complete and Missing Data at Baseline and Follow-ups **eTable 2.** The Incidence of Adverse Health Outcomes According to Baseline Frailty/Nutritional Status
