# Supplementary material for: Association of Frailty and Malnutrition With Long-term Functional and Mortality Outcomes Among Community-Dwelling Older Adults: Results From the Singapore Longitudinal Aging Study 1
Source: JAMA Netw Open. 2018 Jul 13;1(3):e180650. doi: 10.1001/jamanetworkopen.2018.0650 (PMC6324309; doi:10.1001/jamanetworkopen.2018.0650)
Supplement: Supplement. — eTable 1. The Number of Participants With Complete and Missing Data at Baseline and Follow-ups eTable 2. The Incidence of Adverse Health Outcomes According to Baseline Frailty/Nutritional Status [file jamanetwopen-1-e180650-s001.pdf]

Supplementary Online Content

Wei K, Nyunt M-S-Z, Gao Q, Wee S-L, Yap K-B, Ng T-P. Association of frailty and malnutrition with long-term functional and mortality outcomes among community-dwelling older adults: results from the Singapore Longitudinal Aging Study 1. *JAMA Netw Open*. 2018;1(3):e180650. doi:10.1001/jamanetworkopen.2018.0650

eTable 1. The Number of Participants With Complete and Missing Data at Baseline and Follow-ups

eTable 2. The Incidence of Adverse Health Outcomes According to Baseline Frailty/Nutritional Status

This supplementary material has been provided by the authors to give readers additional information about their work.

© 2018 Wei K et al. *JAMA Network Open*.

eTable 1. The Number of Participants With Complete and Missing Data at Baseline and Follow-ups

Characteristics	Baseline (N=2804)		1st Follow-up (N=1849)		2nd Follow-up (N=1332)		Follow-up till March 2017 (N=2804) *	
	Complete	Missing	Complete	Missing	Complete	Missing	Complete	Missing
Frailty score	2760	44	-	-	-	-	-	-
MNA score	2726	78	-	-	-	-	-	-
NSI score	2794	10	-	-	-	-	-	-
IADL/ADL disability	2739	65	1821	28	1305	27	-	-
Quality of life	2722	82	1819	30	1325	7	-	-
Mortality	-	-	-	-	-	-	2552	252

* Only mortality was available in the follow-up till 31 March 2017.

© 2018 Wei K et al. *JAMA Network Open*.

eTable 2. The Incidence of Adverse Health Outcomes According to Baseline Frailty/Nutritional Status

Baseline Frailty/Nutritional Status	IADL/ADL Disability		Poor Quality of Life		Mortality	
	1st Follow-up	2nd Follow-up	1st Follow-up	2nd Follow-up	1st Follow-up	2nd Follow-up
Frailty Status (n, %)	N=1341	N=1003	N=1345	N=993	N=2539	N=2539
Robust	58 (7.76)	42 (7.34)	104 (13.8)	98 (17.2)	18 (1.38)	40 (3.07)
Pre-frailty	77 (13.2)	38 (9.03)	143 (24.7)	115 (27.7)	38 (3.33)	99 (8.67)
Frailty	4 (36.4)	1 (10.0)	6 (50.0)	6 (66.7)	18 (19.4)	31 (33.3)
Nutritional Status (n, %)						
Mini Nutritional Assessment Short-Form	N=1331	N=994	N=1336	N=985	N=2503	N=2503
Normal nutrition (12-14)	84 (9.01)	59 (8.33)	163 (16.8)	141 (19.7)	22 (1.37)	68 (4.24)
At risk of malnutrition (8-11)	49 (13.1)	19 (7.14)	73 (21.2)	69 (27.8)	35 (4.41)	77 (9.70)
Malnourished (0-7)	3 (12.5)	2 (10.0)	12 (50.0)	9 (42.9)	16 (15.2)	23 (21.9)
Nutrition Screening Initiative	N=1354	N=1006	N=1347	N=990	N=2544	N=2544
Good nutrition (0-2)	100 (9.94)	65 (8.72)	154 (15.4)	142 (19.1)	37 (2.10)	95 (5.38)
Moderate nutritional risk (3-5)	37 (12.0)	13 (5.58)	90 (29.2)	65 (30.2)	32 (4.87)	62 (9.44)
High nutritional risk (≥6)	3 (7.50)	3 (10.7)	8 (19.5)	12 (40.0)	7 (5.79)	17 (14.1)

© 2018 Wei K et al. *JAMA Network Open*.
